# Differential Short-Term Repeated Forearm Hyperaemic Reactivity in Coronary Artery Disease Patients Compared to Healthy Low Risk Participants

**DOI:** 10.1155/2012/578504

**Published:** 2012-10-24

**Authors:** Simon L. Bacon, Bernard Meloche, Kim L. Lavoie, André Arsenault

**Affiliations:** ^1^Montreal Behavioural Medicine Centre, Hôpital du Sacré-Cœur de Montréal, A University of Montreal Affiliated Hospital, 5400 Gouin O., Montréal, QC, Canada H4J 1C5; ^2^Montreal Heart Institute, A University of Montreal Affiliated Hospital, 5000 Bélanger East, Montréal, QC, Canada H1T 1C8; ^3^Department of Exercise Science, Concordia University, 7141 Sherbrooke St West, Montréal, QC, Canada H4B 1R6; ^4^Research Centre, Hôpital du Sacré-Cœur de Montréal, A University of Montreal Affiliated Hospital, 5400 Gouin O., Montréal, QC, Canada H4J 1C5; ^5^Department of Psychology, University of Québec at Montréal (UQAM), P.O. Box 8888, Succursale Centre-Ville, Montréal, QC, Canada H3C 3P8

## Abstract

The hyperaemic response of the forearm is a widely used technique to assess the vascular reactivity. Little is known about the short-term reproducibility and the possible exhaustion of this response in normal or diseased states. As such, the current study was conducted to assess this phenomenon using a unique nuclear medicine- (NM-) based technique. 19 patients with coronary artery disease (CAD) undergoing NM exercise stress tests and 15 low risk (LR) participants completed 2 reactive hyperaemia tests, using a SPECT-based technique, separated by 15  min. Analyses revealed that CAD patients had lower hyperaemic responses than LR participants (*P* < .001), and that there was a significant group × time interaction (*P* < .005), such that LR participants showed a larger decrease in the reactivity (5.2 ± 0.4 to 3.6 ± 0.4) than the CAD patients (2.9 ± 0.3 to 2.6 ± 0.3). These results suggest that there is a variability, due to disease states, in the reproducibility of the hypaeremic reactivity. This needs to be taken into account in short-term repeated measure studies.

## 1. Introduction

Endothelial dysfunction is thought to be a major factor in the development and progression of coronary artery disease (CAD) [1]. Over the past several years, measures of endothelial function have become widely used in medical research. One such technique that has gained popularity is the occlusion-induced ischemia of the forearm using a blood pressure cuff. Release of the ischemia induces a hyperaemic reactivity measurable in the brachial artery [1, 2]. This assessment technique has been shown to have reasonable [2–5], though not perfect [6, 7], long-term reproducibility (e.g., day-to-day or month-to-month). However, little is known about the short-term (15 min) reproducibility of hyperaemic reactivity, or if this reproducibility is different between patients with CAD and healthy controls. Given the desirability of being able to conduct multiple tests over a short period of time for use in clinical trials and the assessment of acute exposures (e.g., exercise, acute psychological stress, pharmacological) such information would be important to be known. We previously compared hyperaemic reactivity in CAD patients and low risk participants using a nuclear medicine based forearm hyperaemic reactivity technique (FHR) [3]. This measure has very good discriminant properties and good day-to-day reproducibility. However, its short-term reproducibility is unknown and, as such, the objectives of the current study were to test this reproducibility in a sample of CAD patients and low risk controls.

## 2. Materials and Methods

### 2.1. Participants

A total of 19 patients referred for single photon emission computed tomography (SPECT) exercise stress tests in the Nuclear Medicine Service of the Montreal Heart Institute and 15 low risk participants from the EPIC Training Centre (a health fitness facility affiliated with the Montreal Heart Institute) were included. To be eligible all participants had to be over 40 years old and speak either English or French, and, for women, they had to have reached menopause but not be taking any hormone replacement therapy. Low risk participants were excluded if they had any history of CAD or were taking any cardiac medication. Patients were defined as having CAD if they had at least one of the following: surgical or angioplasty revascularization, acute coronary syndrome, or angiographic or myocardial scintigraphic defined significant CAD. However, patients were excluded if they had experienced a cardiac event in the last 2 weeks (e.g., acute coronary syndrome) or had a more prominent medical condition than CAD (e.g., cancer chronic obstructive pulmonary disease) or if there was a differential blood pressure greater than 20 mmHg between the right and left arms. All participants provided standard demographic, medical history, and medications details. Current blood pressure and lipids were also recorded. The study was approved by the Human Ethics Committee of the Montreal Heart Institute and a written informed consent was obtained from all participants.

### 2.2. Brachial Artery Reactivity

 All participants underwent 2 successive forearm hyperaemic reactivity (FHR) evaluations 15 minutes apart, the details of which have been published previously [3]. In brief, participants were seated with their arms placed over the top of a large field-of-view gamma camera with palms placed on the camera. A blood pressure cuff was placed on the right arm with a catheter placed in the left arm. The blood pressure cuff was inflated to 50 mm Hg above systolic pressure for 5 min. Thirty seconds after cuff release a bolus of technetium (Tc99m) tetrofosmin was injected (0.42 mCi/kg for CAD patients and 0.21 mCi/kg for the LR group). This meant that with a 30-second transit time the tracer was present in the 2 arms approximately 60 seconds after cuff release, the point of maximal secondary vasodilation. Dynamic images were acquired at a sampling rate of 1 frame per second using a 128 × 128 matrix during the whole process and 10 minutes following cuff release. Data were derived from gamma-camera first-pass activity-time curves (ATC). The relative uptake ratio (RUR) of the hyperemic compared to the nonhyperemic arm was used to define the brachial artery reactivity. This measure has been shown to predict CAD [3, 8], has a high day-to-day test-retest reliability (*r* = .89) [9] and excellent inter- and intrarater reliability (*r* = .98) [10], and is consistent with similar nuclear medicine based techniques [11]. All testing was conducted in the morning following a minimum of 8-hour fast.

### 2.3. Statistical Analyses

Demographic and medical comparisons between CAD patients and LR participants were made using unpaired *t*-test (continuous data) or chi-squared test (categorical data). Several techniques were used to assess the effect of repeated FHR testing in both groups. Initially, a repeated measure general linear model was conducted, with group as the between-subject variable and time as the repeated element. Secondly, we conducted separate intraclass correlations between the repeated RUR results for the 2 groups. Finally, we created Bland-Altman plots for the 2 groups to assess consistency in the RUR findings.

## 3. Results

Details of the baseline demographics of the CAD patients and low risk participants are reported in Table 1. Unsurprisingly, CAD patients were more likely to be current smokers, have diabetes, and have hypertension. In addition, patients had higher fasting glucose levels and lower HDL levels. In contrast, low risk participants had higher fasting cholesterol and LDL levels. The difference in lipid levels may in part be accounted for by the fact that nearly 70% of CAD patients were taking lipid lowering therapy.

**Figure 1:**
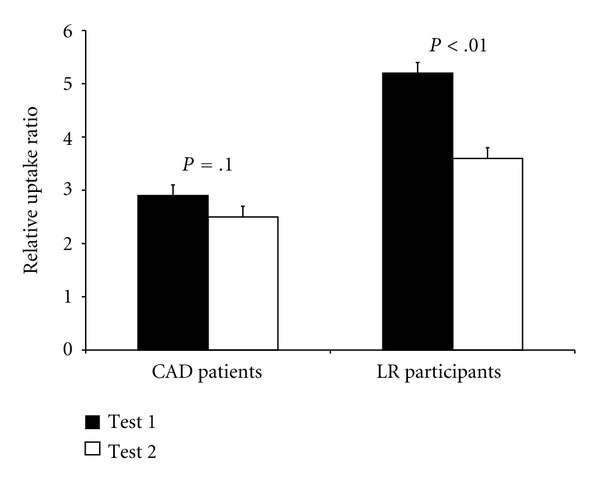
Mean and SEM relative uptake ratio (RUR) values for coronary artery disease (CAD) patients and low risk (LR) participants for test 1 and test 2.

**Figure 2:**
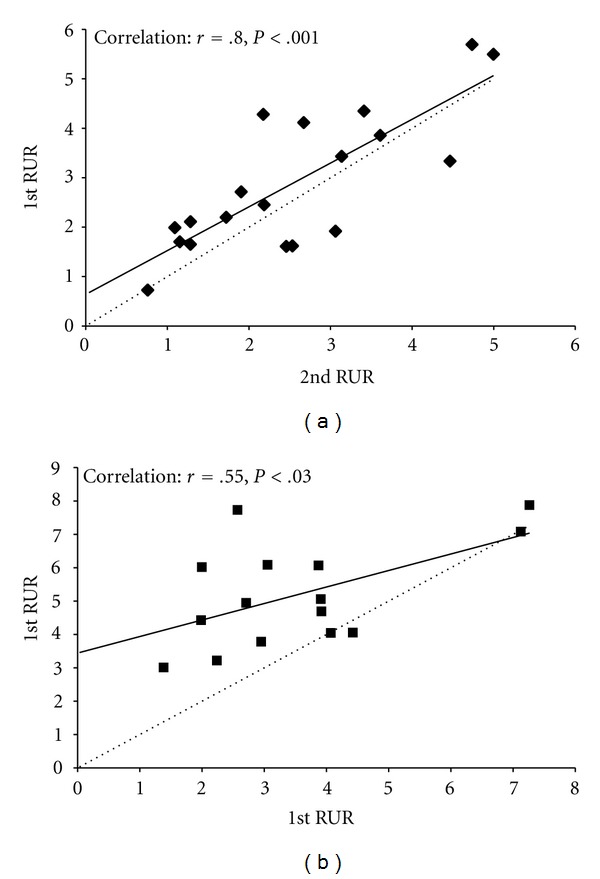
Plots of short-term reproducibility for (a) CAD patients (◆) and (b) LR participants (■). Solid lines indicate the regression line for the correlation and the dotted line indicates perfect reproducibility.

**Figure 3:**
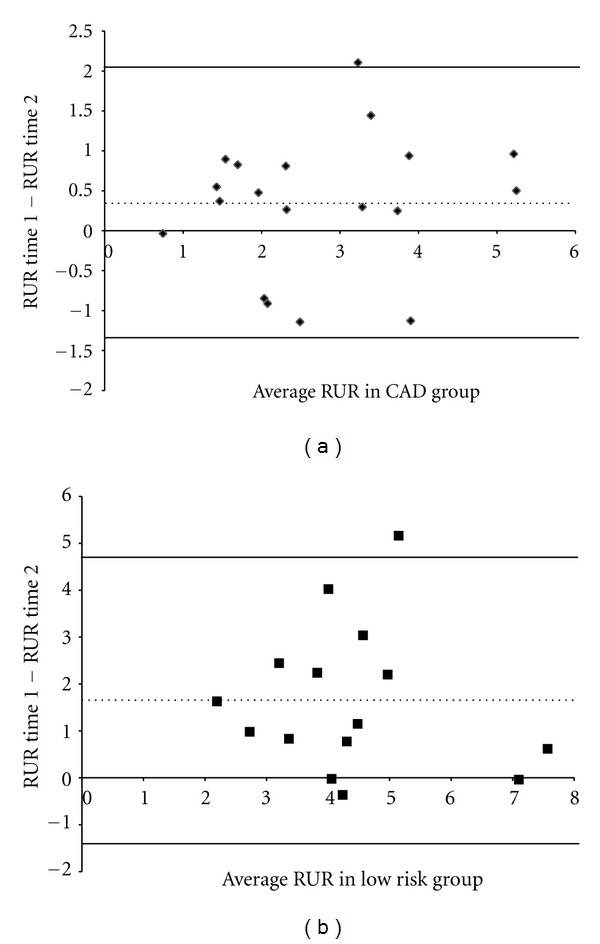
Bland-Altman plots of consistency for the RUR measures in (a) CAD patients (◆) and (b) LR participants (■). Solid lines indicate the 95% CI's and the dotted line indicates the mean difference.

As shown in Figure 1, analyses revealed a main effect of group, such that CAD patients had lower RUR than low risk participants (mean ± SEM = 2.7 ± 0.2 versus 4.4 ± 0.3,  *F* = 12.6,  *P* < .001), as well as a main effect of time, where RUR was lower on the 2nd test compared to the 1st test (3.1 ± 0.3 versus 4.1 ± 0.3,  *F* = 22.4,  *P* < .001). There was a significant group × time interaction (*F* = 9.5, *P* = .004) such that low risk participants showed a larger decrease in RUR from test 1 to test 2 (5.2 ± 0.4 to 3.6 ± 0.4, *F* = 16.62, *P* = .001) than the CAD patients (2.9 ± 0.3 to 2.6 ± 0.3, *F* = 3.08, *P* = .096). Intra-class correlational analyses revealed a significant correlation between test 1 and test 2 for both CAD patients (*r* = .80, *P* < .001) and LR participants (*r* = .55, *P* = .03). However, linear regression models showed a remarkable reproducibility between tests with no significant exhaustion for CAD patients (slope = .89, intercept = 0.6, *t* = 1.4, *P* = .19), whereas low risk participants had a systematically lower second reading (slope = .55, intercept = 3.4, *t* = 4.2,  *P* = .001) (Figure 2). As shown in Figure 3, Bland-Altman plots found consistency between the 2 RUR readings in the CAD patients (mean difference = 0.35, 95% CI's = −1.34–2.04), but not in the LR participants (mean difference = 1.64, 95% CI's = −1.42–.70). To ensure that the response seen was not just due to the presence of the increase in blood flow from the first examination in the LR participants we conducted a repeated measures GLM on the slopes of the control arm. This analysis found no effect of time (*F* = 0.6, *P* = .455) nor a group × time interaction (*F* = 2.4, *P* = .135), thus suggesting that there was no carry-over effect that was specific to the LR group.

## 4. Discussion

To our knowledge, this is the first study to directly compare the short-term reproducibility of brachial artery testing in older CAD patients and age matched low risk controls. As with our previously reported data [3], for the 1st hyperaemic reactivity test, low risk participants had significantly higher RURs compared to CAD patients, and the difference was consistent with our previously published cut point of 3.55. However, there was no difference between groups when the ischemic challenge was repeated 15 minutes later, indicating that low risk participants showed a significant drop in RUR whereas the CAD patients did not. The results also indicated that the 15-minute test-retest reliability of the FHR technique is adequate for CAD patients but not for low risk age matched healthy participants. 

The results seen in the current study for the CAD patients are consistent with a recent report which showed that the 30-minute test-retest reliability of flow-mediated dilatation in patients with CAD was high with a variation of around 10% (consistent with our study) [12]. In contrast to our results, a study of young healthy individuals found that flow-mediated dilatation did not change when repeated at 5 or 15 minutes and showed excellent short-term test-retest reliability [13]. One key difference which may explain this inconsistency between the Barton paper and ours is the age of the population under investigation. Our low risk population had a mean age of 59 compared to a mean age of 27 in the Barton study. As we know, aging has a significant effect on brachial artery reactivity, independent of any disease development [14–16], as such, the capacity of an older healthy artery to return to resting levels may be reduced compared to a young healthy artery. Caution must also be taken in comparing the flow-mediated dilatation and FHR techniques as they do differ on a number of important points, specifically the fact that the control measure is made concurrently in the FHR (compared to before hyperaemia in flow-mediated dilatation) and the FHR has greater inter- and intrarater reliability than flow-mediated dilatation. Both of these are potentially important when determining test-retest reliability. That being said, it is possible that the phenomenon seen in the current study could be a function of the nature of the FHR test and only simultaneous assessment of its test-retest reliability with other measures of brachial artery reactivity would be able to determine if this is true.

Though the current study was not designed to explore the underlying mechanisms of the differences seen, there are several possible explanations. It is believed that a basic physiological underpinning of the reactive hyperaemic response is derived from increased shear stress, generated by the reintroduced blood flow, provoking the endothelium to release nitric oxide (NO) thus leading to vasodilatation [1]. A number of studies have detailed a reduced response between patients with CAD and healthy participants [17–19], and, as such, the fact that the current study found a lower RUR in CAD patients compared to low risk participants for the initial test was unsurprising. In contrast, during the repeat test the low risk participants were not able to match their previous vasodilatory response. This apparent reduction in capacity could result from a reduced capacity to produce NO [20], a reduced sensitivity of smooth muscle cells to released NO [21], or the production of counterregulatory vasoconstricting factors (e.g., endothelin [22]). The observation that the low risk participants had a significantly higher response during the first test compared to the CAD patients might suggest that they have an increased capacity to produce nitric oxide with an incapacity to repeat the response following a short restorative period. Conversely, the CAD patients, who had a blunted response to the initial hyperaemia, may not require a long restorative period and as such have a similar blunted response during the second hyperaemia. Further work is needed to discern which one or a combination of these mechanisms is driving the lack of dilation during the second test in healthy participants compared to CAD patients. 

From a clinical perspective, the current results provide a “starting point” to further explore the impact of short-term repeated hyperemia response and what may be happening to generate a differential response in those with CAD and at LR. It may be that such changes could be associated with greater future risk. However, more work is needed to be able to confirm the current result, detail potential mechanisms leading to this phenomenon, and assess the predictive utility of such a finding.

In summary, the current study suggests that low risk participants have reduced hyperaemic responses after short-term repeated hyperaemic challenges, possibly indicating some kind of “exhaustion” phenomenon. In contrast, CAD patients, who had lower baseline hyperaemic reactivity, displayed a high level of short-term reproducibility. These results need to be taken into account when utilising this technique in future studies.

## Figures and Tables

**Table 1:** Demographic characteristics for CAD patients and LR participants.

	CAD patients	Low risk participants	*t*/*χ* ^2^	*P*
Number	19	15		
Age (years)	62 ± 11	59 ± 9	0.76	.45
Men (%)	89%	100%	1.68	.20
BMI (kg/m^2^)	29.7 ± 3.8	27.4 ± 4.0	1.72	.10
Cholesterol (mmol/L)	4.5 ± 1.1	5.2 ± 0.9	2.17	.04
LDL (mmol/L)	2.6 ± 1.1	3.3 ± 0.7	2.30	.03
HDL (mmol/L)	1.1 ± 0.2	1.3 ± 0.3	2.15	.04
Triglycerides (mmol/L)	1.8 ± 0.8	1.4 ± 0.9	1.47	.15
Glucose (mmol/L)	6.5 ± 2.1	5.3 ± 0.5	2.17	.04
Resting SBP (mmHg)	135 ± 30	128 ± 12	0.88	.39
Resting DBP (mmHg)	71 ± 10	74 ± 14	0.73	.47
Taking aspirin (%)	74%	0%	23.93	<.01
Taking beta blocker	59%	0%	15.98	<.01
Taking calcium channel blocker (%)	37%	0%	8.48	<.01
Taking ACE inhibitor	42%	0%	10.11	<.01
Taking lipid lowering medication (%)	68%	0%	20.99	<.01
Current smokers (%)	16%	0%	2.60	.11
Have diabetes (%)	38%	0%	5.75	.02
Have hyperlipidemia (%)	74%	0%	29.00	<.01
Have hypertension (%)	56%	0%	9.66	<.01
Family history of CAD (%)	37%	13%	2.38	.12

BMI: body mass index; LDL: low density lipoproteins; HDL: high density lipoproteins; SBP: systolic blood pressure; DBP: diastolic blood pressure; CAD: coronary artery disease.
